# Adipose Tissue and Brain Metabolic Responses to Western Diet—Is There a Similarity between the Two?

**DOI:** 10.3390/ijms21030786

**Published:** 2020-01-25

**Authors:** Arianna Mazzoli, Maria Stefania Spagnuolo, Cristina Gatto, Martina Nazzaro, Rosa Cancelliere, Raffaella Crescenzo, Susanna Iossa, Luisa Cigliano

**Affiliations:** 1 Department of Biology, University of Naples Federico II, 80134 Naples, Italy; arianna.mazzoli@unina.it (A.M.); crigatto51@gmail.com (C.G.); martinanazzaro52@gmail.com (M.N.); cancelliererosa@gmail.com (R.C.); rcrescen@unina.it (R.C.); 2 Department of Bio-Agrofood Science, Institute for the Animal Production System in Mediterranean Environment, National Research Council Naples (CNR-ISPAAM), 80147 Naples, Italy; mariastefania.spagnuolo@cnr.it

**Keywords:** adipose tissue, hippocampus, frontal cortex, adiponectin, haptoglobin, lipocalin, BDNF, synaptic proteins

## Abstract

Dietary fats and sugars were identified as risk factors for overweight and neurodegeneration, especially in middle-age, an earlier stage of the aging process. Therefore, our aim was to study the metabolic response of both white adipose tissue and brain in middle aged rats fed a typical Western diet (high in saturated fats and fructose, HFF) and verify whether a similarity exists between the two tissues. Specific cyto/adipokines (tumor necrosis factor alpha (TNF-α), adiponectin), critical obesity-inflammatory markers (haptoglobin, lipocalin), and insulin signaling or survival protein network (insulin receptor substrate 1 (IRS), Akt, Erk) were quantified in epididymal white adipose tissue (e-WAT), hippocampus, and frontal cortex. We found a significant increase of TNF-α in both e-WAT and hippocampus of HFF rats, while the expression of haptoglobin and lipocalin was differently affected in the various tissues. Interestingly, adiponectin amount was found significantly reduced in e-WAT, hippocampus, and frontal cortex of HFF rats. Insulin signaling was impaired by HFF diet in e-WAT but not in brain. The above changes were associated with the decrease in brain derived neurotrophic factor (BDNF) and synaptotagmin I and the increase in post-synaptic protein PSD-95 in HFF rats. Overall, our investigation supports for the first time similarities in the response of adipose tissue and brain to Western diet.

## 1. Introduction

Lifestyle, nutrition and lack of physical exercise is increasing the number of overweight or obese people in the global population. The increased consumption of sugar- and fat-rich industrial foods, which are cheaper and easily available, contributes to the accumulation of peripheral and/or visceral adipose tissue [1]. In the last decade, dietary fats and sugars were also identified as risk factors for cognitive decline and neurodegeneration, through altered brain metabolism, neuroinflammation and neuronal dysfunction [2,3,4,5,6].

The increased risk for obese individuals to develop brain disease might be due to the capacity of adipose tissue to communicate with the brain and impact its function. Therefore, the analysis of specific markers, whose adipose and/or brain levels are affected by diet, might contribute to unveil the intersection between obesity and neurodegeneration. In particular, proinflammatory cytokines, namely tumor necrosis factor alpha (TNFα), are implicated in the development of neuronal insulin resistance [7,8]. Since both cytokines and insulin in the brain regulate synaptic plasticity, learning and memory, neuroinflammation and neuronal insulin resistance have been proposed to be involved in obesity-associated brain impairment [9].

Middle-age, an earlier stage of the aging process, is a life phase in which humans and animals are more prone to develop diet-induced metabolic alterations. In fact, overweight is especially prevalent in the middle-aged population (40.2%) compared to younger or older adults (32.3% and 37.0%, respectively) [10]. In addition, overweight during midlife has been associated to a higher risk of developing cognitive disorders, including Alzheimer’s disease [11,12]. Accordingly, we recently found that short-term dietary treatment with a high fat-high fructose diet was able to elicit inflammation and oxidative stress in plasma and brain of middle-aged rats [6,13]. We therefore decided to investigate the possible similarity in the response of white adipose tissue and brain in middle aged rats that were fed a typical Western diet (high in saturated fats and fructose, HFF) for 4 weeks. In particular, specific cyto/adipokines (TNF-α, adiponectin), critical obesity-inflammatory markers (haptoglobin, lipocalin), and insulin signaling or survival protein network (insulin receptor substrate 1 (IRS), Akt, Erk) were measured in both adipose tissue and hippocampus/frontal cortex, two key areas for learning and memory. Moreover, in order to further highlight whether the HFFdiet is associated with an impairment of brain function, we evaluated brain-derived neurotrophic factor (BDNF) and its receptor (Tropomyosin receptor kinase B, TrkB), as well as specific pre- and post-synaptic proteins in the hippocampus and frontal cortex, known to be susceptible to nutritional stimuli.

## 2. Results

### 2.1. Metabolic Characterization

**Figure 1:**
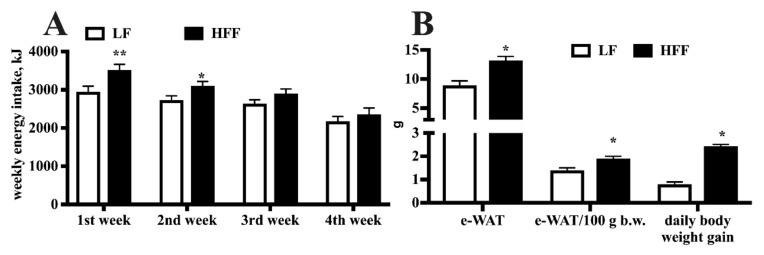
Weekly energy intake (**A**), epididymal white adipose tissue (e-WAT) weight and body weight gain (**B**) in middle-aged rats fed low fat (LF) or high fat-high fructose (HFF) diet for four weeks. Values are the means ± SEM of eight rats. * *p* < 0.05, ** *p* < 0.01 compared to low-fat diet (two-way ANOVA followed by Tukey post-test for energy intake data and two-tailed Student’s t-test for e-WAT weight and daily body weight gain data).

Dietary administration of HFF diet elicited caloric hyperphagia in the first and, less markedly, in the second week, while during the third and fourth week the caloric intake was similar in the two groups of rats (Figure 1A). In addition, HFF rats exhibited significantly higher body weight gain, as well as higher body content of epididymal white adipose tissue (e-WAT), both as absolute amount and as % of body weight (Figure 1B).

**Figure 2:**
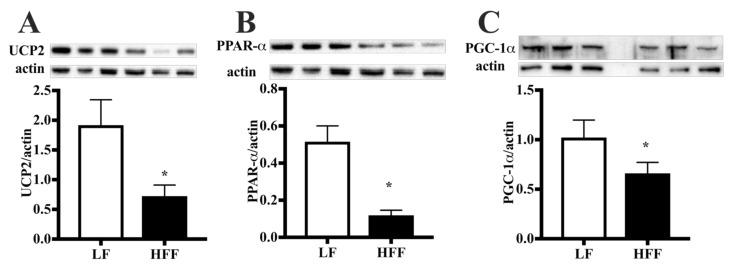
Protein content (with representative western blot) of uncoupling protein 2 (UCP2) (**A**), peroxisome proliferator activated receptor alpha (PPAR-α) (**B**) and peroxisome proliferator-activated receptor gamma coactivator 1-alpha (PGC-1α) (**C**) in epididymal white adipose tissue (e-WAT) from middle-aged rats fed low fat (LF) or high fat-high fructose (HFF) diet for four weeks. Values are the means ± SEM of eight rats. * *p* <0.05 compared to low-fat diet (two-tailed Student’s t-test).

Key proteins involved in the regulation of adipocyte function, namely uncoupling protein 2 (UCP2), peroxisome proliferator-activated receptor alpha (PPAR-α), and peroxisome proliferator-activated receptor gamma coactivator 1-alpha (PGC-1α), were found to be downregulated in HFF rats compared to rats fed low fat (LF) diet (Figure 2).

### 2.2. Inflammatory Markers

**Figure 3:**
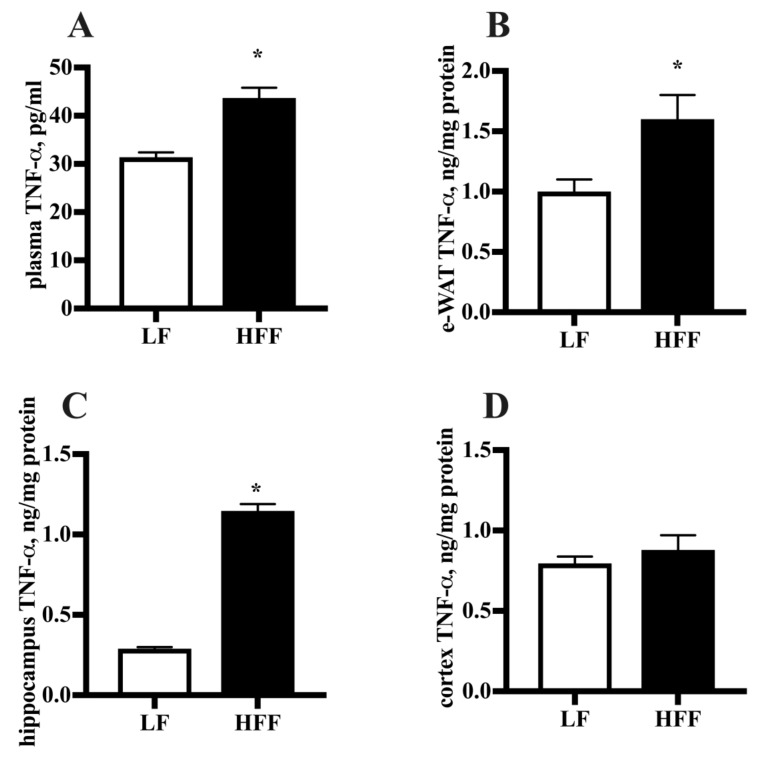
Levels of tumor necrosis factor alpha (TNF-α) detected by enzyme-linked immunosorbent assayin plasma (**A**), epididymal white adipose tissue (e-WAT) (**B**), hippocampus (**C**), and frontal cortex (**D**) from middle-aged rats fed low fat (LF) or high fat-high fructose (HFF) diets for four weeks. Values are the means ± SEM of eight rats. * *p* < 0.05 compared to low-fat diet (two-tailed Student’s t-test).

**Figure 4:**
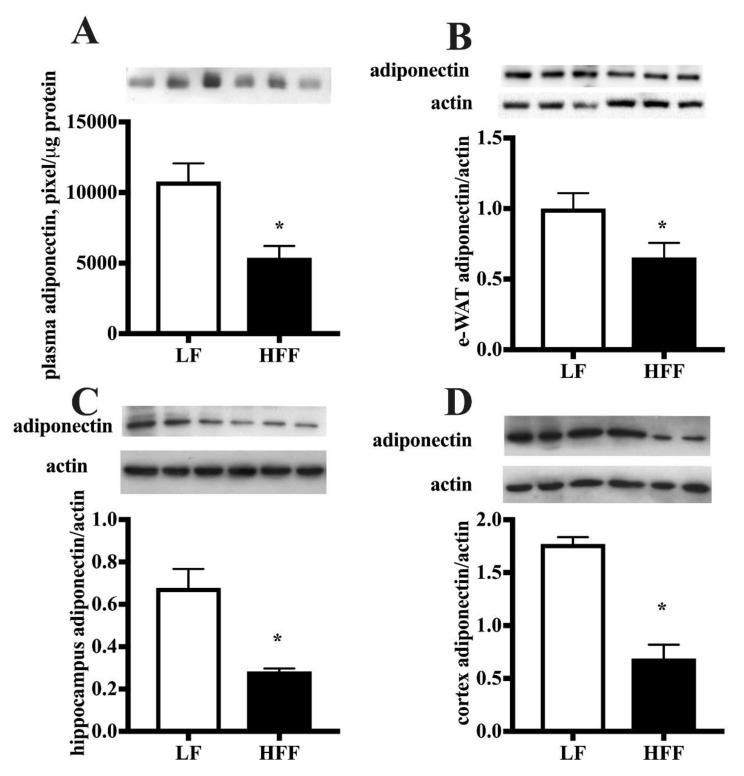
Protein content (with representative western blot) of adiponectin in plasma (**A**), epididymal white adipose tissue (e-WAT) (**B**), hippocampus (**C**), and frontal cortex (**D**) from middle-aged rats fed low fat (LF) or high fat-high fructose (HFF) diets for four weeks. Plasma adiponectin was detected by using 5 μL of sample. Values are the means ± SEM of eight rats. * *p* < 0.05 compared to low-fat diet (two-tailed Student’s t-test).

Since increased intake of fat and fructose is associated with systemic and tissue inflammation [14,15], we wanted to verify whether this response was elicited after our dietary treatment. To this end, we assessed markers of inflammation in plasma, e-WAT and brain areas. TNF-α levels were significantly increased in plasma, e-WAT and hippocampus of HFF rats compared to controls, while no variation was found in frontal cortex (Figure 3). Furthermore, we titrated the level of adiponectin, an adipokine which plays a role in the reduction of oxidative stress and inflammatory cascade [16] and was also reported to exert neuroprotective effect [17]. Adiponectin amount was found significantly reduced in plasma, e-WAT, hippocampus, and frontal cortex of HFF fed rats (Figure 4).

**Figure 5:**
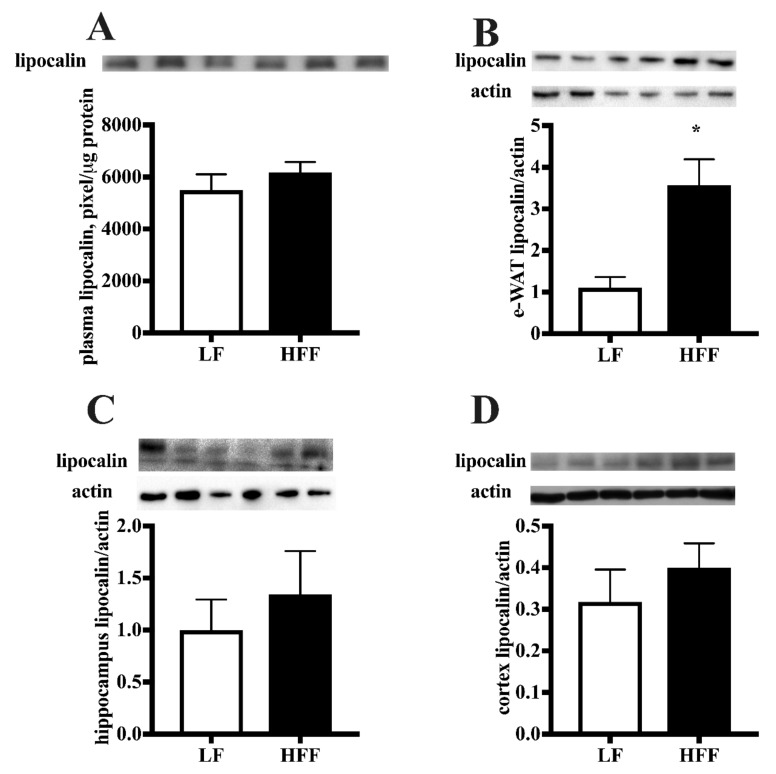
Protein content (with representative western blot) of lipocalin in plasma (**A**), epididymal white adipose tissue (e-WAT) (**B**), hippocampus (**C**), and frontal cortex (**D**) from middle-aged rats fed low fat (LF) or high fat-high fructose (HFF) diet for four weeks. Values are the means ± SEM of eight rats. * *p* < 0.05 compared to low-fat diet (two-tailed Student’s t-test).

**Figure 6:**
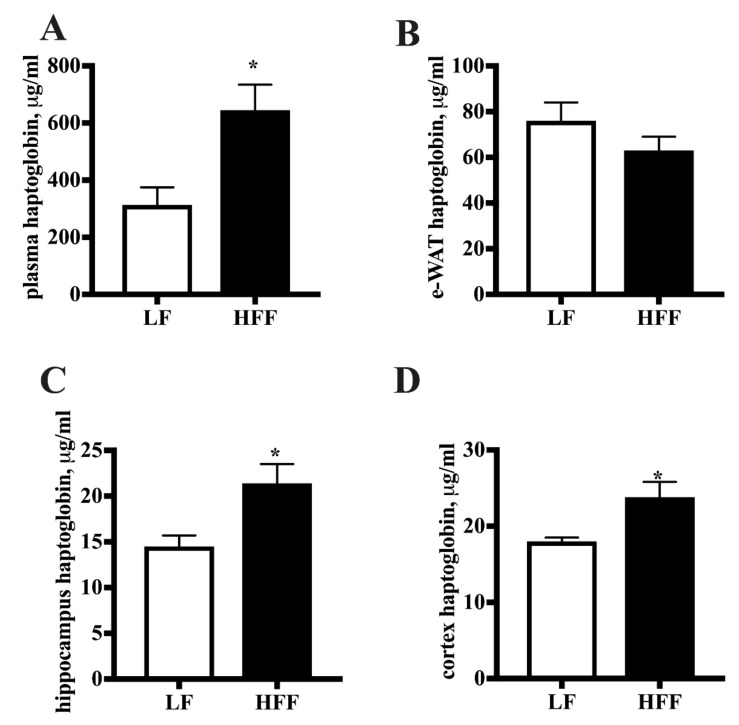
Levels of haptoglobin detected by enzyme-linked immunosorbent assay in plasma (**A**), epididymal white adipose tissue (e-WAT) (**B**), hippocampus (**C**), and frontal cortex (**D**) from middle-aged rats fed low fat (LF) or high fat-high fructose (HFF) diet for four weeks. Values are the means ± SEM of eight rats. * *p* < 0.05 compared to low-fat diet (two-tailed Student’s t-test).

The level of haptoglobin and lipocalin, which are considered markers of both inflammation and adiposity [18,19], was measured. As shown in Figure 5, no diet-associated variation of lipocalin concentration was detected in plasma, hippocampus or cortex, while in e-WAT lipocalin protein content significantly increased in HFF rats. Conversely, haptoglobin level was found increased, following HFF diet, in plasma, frontal cortex, and hippocampus, while no changes were detected in e-WAT (Figure 6).

### 2.3. Insulin Signaling

**Figure 7:**
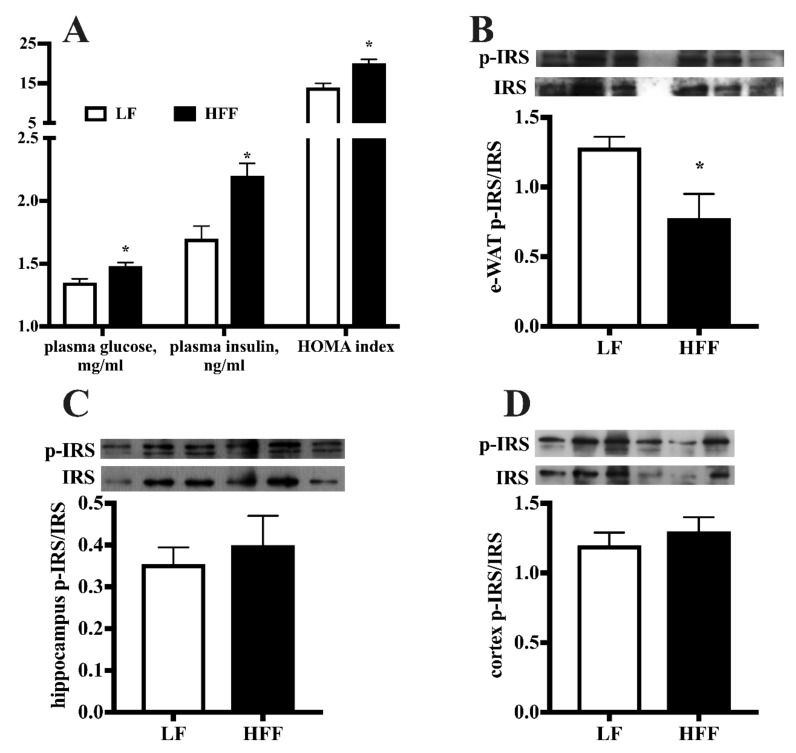
Plasma levels of glucose and insulin and Homeostatic model assessment (HOMA) index (**A**), protein content (with representative western blot) of phosphorylated Insulin Receptor Substrate (p-IRS) in epididymal white adipose tissue (e-WAT) (**B**), hippocampus (**C**), and frontal cortex (**D**) from middle-aged rats fed low fat (LF) or high fat-high fructose (HFF) diet for four weeks. Values are the means ± SEM of eight rats. * *p* < 0.05 compared to low-fat diet (two-tailed Student’s t-test).

Plasma metabolic profile evidenced that the HFF diet was able to induce systemic insulin resistance, with higher fasting glucose and insulin levels and increased Homeostasis model assessment (HOMA) index (Figure 7A). At the tissue level, insulin signaling was impaired by HFF diet in e-WAT, as shown by a significant decrease in the activatory phosphorylation of IRS (Figure 7B), while this effector was not affected by diet in hippocampus (Figure 7C) and cortex (Figure 7D).

**Figure 8:**
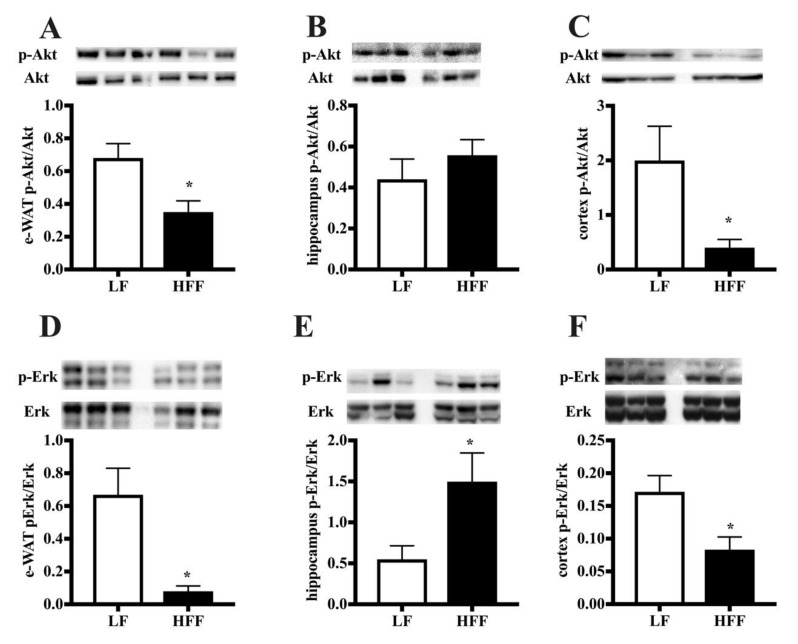
Protein content (with representative western blot) of phosphorylated Akt (p-Akt) and Erk (p-Erk) in epididymal white adipose tissue (e-WAT) (**A**,**D**), hippocampus (**B**,**E**), and frontal cortex (**C**,**F**) from middle-aged rats fed low fat (LF) or high fat-high fructose (HFF) diets for four weeks. Values are the means ± SEM of eight rats. * *p* < 0.05 compared to low-fat diet (two-tailed Student’s t-test).

The analysis of downstream effectors of IRS, namely Akt and Erk, revealed an impairment for both effectors in e-WAT (Figure 8A,B). The activation of the Akt and Erk pathway was found significantly decreased by HFF diet in frontal cortex (Figure 8E,F), while in hippocampus was evident a significant increase in the Erk activation (Figure 8D), with no variation in the Akt pathway (Figure 8C).

### 2.4. BDNF and TrkB

**Figure 9:**
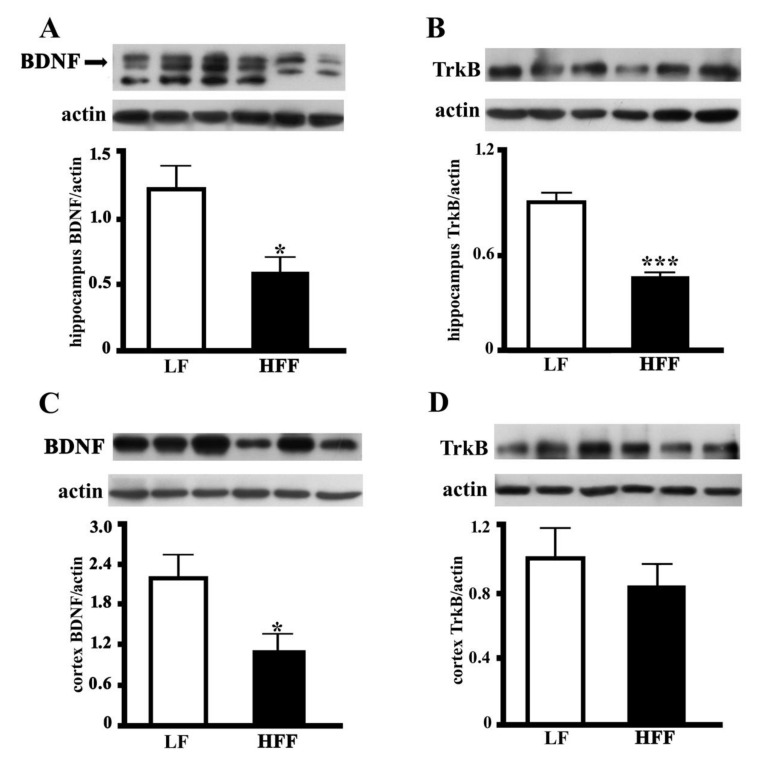
Protein content (with representative western blot) of brain derived neurotrophic factor (BDNF) and tropomyosin receptor kinase b (TrkB) levels in hippocampus (**A**,**B**), and frontal cortex (**C**,**D**) from middle-aged rats fed low fat (LF) or high fat-high fructose (HFF) diets for four weeks. Values are the means ± SEM of eight rats. * *p* < 0.05 compared to low-fat diet (two-tailed Student’s t-test).

BDNF is a key cerebral factor involved in a wide range of neurophysiological processes and has a multipotent impact on brain signaling and synaptic plasticity [20]. The BDNF level was measured in both the hippocampus and frontal cortex, and a significant diet dependent decrease was observed (Figure 9A,C). We also analyzed adipose tissue samples for the presence of BDNF, but we detected only a very faint band (data not shown). At present, we cannot say whether this very faint band reflects a small amount of BDNF produced locally in e-WAT or a small amount coming from the plasma. Further work is required to discriminate between the two options. We further investigated whether the dietary treatment could affect the amount of TrkB receptor, as BDNF activities, such as enhancement of synaptic plasticity, neuroprotection, and stimulation of neuronal fibers growth, are mediated by neurotrophin binding to this receptor [20]. As shown in Figure 9B, TrkB level was significantly lower in the hippocampus of HFF rats, while it was not affected by diet in the frontal cortex (Figure 9D).

### 2.5. Synaptic Proteins in the Hippocampus and Frontal Cortex

**Figure 10:**
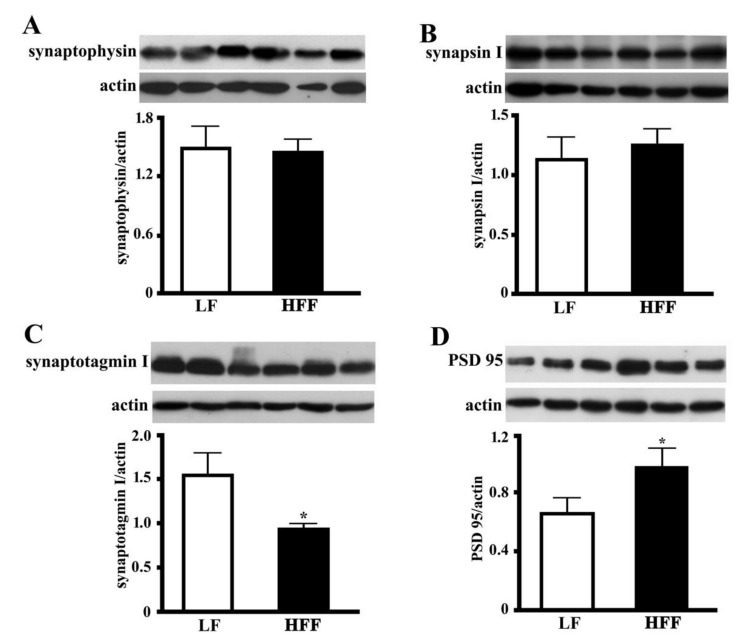
Protein content (with representative western blot) of presynaptic proteins synaptophysin (**A**), synapsin I (**B**) and synaptotagmin I (**C**), and postsynaptic protein PSD-95 (**D**) in the hippocampus from middle-aged rats fed low fat (LF) or high fat-high fructose (HFF) diets for four weeks. Values are the means ± SEM of eight rats. * *p* < 0.05 compared to low-fat diet (two-tailed Student’s t-test).

**Figure 11:**
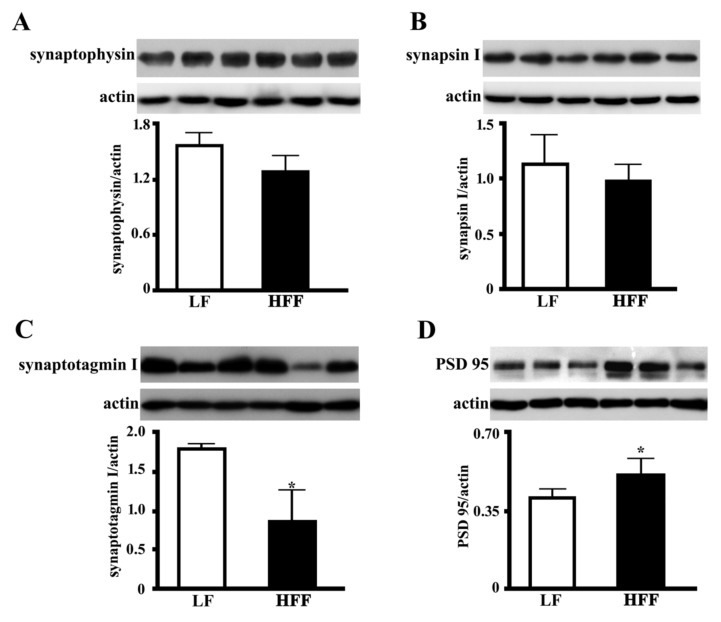
Protein content (with representative western blot) of presynaptic proteins synaptophysin (**A**), synapsin I (**B**) and synaptotagmin I (**C**), and postsynaptic protein PSD-95 (**D**) in the frontal cortex from middle-aged rats fed low fat (LF) or high fat-high fructose (HFF) diets for four weeks. Values are the means ± SEM of eight rats. * *p* < 0.05 compared to low-fat diet (two-tailed Student’s t-test).

In order to clarify the impact of HFF diet on key markers of synaptic function, the levels of presynaptic proteins synaptophysin, synapsin I and synaptotagmin I, and post synaptic density protein 95 (PSD-95) were measured in hippocampus and frontal cortex of HFF and control rats. Synaptophysin and synapsin I are involved in synaptic growth, as they play key roles in synapse formation, maturation, and maintenance [21]. Synaptotagmin I is a major calcium sensor for transmitter release at central synapse and is also crucial for clamping synaptic vesicle fusion in mammalian neurons [22]. HFF diet did not affect synapsin I or synaptophysin level, while it was associated with a marked decrease of synaptotagmin both in the hippocampus (Figure 10) and the frontal cortex (Figure 11). In the next step, we examined the level of PSD-95, a key protein for the function of neurotransmitter receptors [23]. Interestingly, we found that the expression PSD-95 was significantly increased in hippocampus (Figure 10) and frontal cortex (Figure 11) of HFF rats.

## 3. Discussion

The risk of brain dysfunction has been shown to be increased in overweight or obese subjects [24,25,26]. The underlying mechanisms are not precisely defined, but obesity has been associated with several processes related to the acceleration of aging, including oxidative stress and inflammation [27,28]. The link between obesity and brain disease can be further clarified by studying the alteration of adipose tissue physiology, concomitantly with brain modifications in these conditions. In fact, it has been suggested that the molecules released or produced by the adipose tissue could be the molecular link between obesity and brain dysfunction [29]. Indeed, expansion of adipose tissue leads to local inflammation and release of cytokines and adipokines into the systemic circulation, which in turn could contribute to the pathogenesis of brain disorders [30,31]. Since this issue has not been investigate in depth, we sought to investigate the effect of a short-term Western diet on white adipose tissue functional markers and their possible similarity with changes in two critical brain areas for learning and memory, namely, the hippocampus and frontal cortex.

Metabolic phenotyping of middle-aged rats evidenced the deleterious effects of HFF diet, with the increase in the absolute and relative weight of e-WAT. This adipose depot is among the largest and most readily accessible fat pads in the rat [32], and has been shown to reach its maximum expansion earlier (after 4 weeks of high fat feeding) than other adipose tissue depots [33]. The expansion of e-WAT in HFF rats well correlates with the increased caloric intake during the first two weeks and with the downregulation in the content of key proteins involved in the regulation of adipocyte function, namely UCP2, PPAR-α, and PGC-1α. In fact, PPARα and PGC-1α play a central role in the metabolic regulation in adipose tissue [34,35]. In addition, UCP2 may influence systemic metabolism by regulating the release of adipokines by this tissue [36]. Interestingly, we recently reported a similar decrease in UCP2, PPAR-α, and PGC-1α in brain regions of middle-aged rats fed the same Western diet [6].

HFF diet elicited whole-body insulin resistance. The same metabolic impairment was evident also in e-WAT, where downstream effectors of insulin signaling (IRS, Akt, and Erk) were less activated in HFF rats, thus suggesting a condition of insulin resistance in this tissue.

Systemic inflammation was also found in HFF rats, as demonstrated by the increase in plasma levels of both TNF-α and haptoglobin, which is one of the most represented acute-phase proteins. Similarly, e-WAT from HFF rats exhibited increased TNF-α content, in agreement with the frequent association between insulin resistance and inflammation [37]. The above increase is of relevance and could be at the basis of increased lipocalin content found in e-WAT from these animals. In fact, lipocalin expression in adipocytes is regulated by obesity and TNF-α and it can, in turn, induce insulin resistance [38]. In line with the systemic and adipose inflammatory status, the here presented results show that a condition of brain inflammation occurs in response to the HFF diet. As a matter of fact, a diet-associated increase in TNF-α was observed in hippocampus, and haptoglobin level was found higher both in hippocampus and frontal cortex of HFF rats. It is worth mentioning that no data are available thus far on diet-associated changes of haptoglobin in brain of middle-aged rats. The increase in brain haptoglobin might represent a protective mechanism against the condition of enhanced oxidative stress found in hippocampus and frontal cortex [6], due to its well-known antioxidant activity [39,40].

The above inflammatory status might contribute to the onset of brain insulin resistance, however, interestingly, we did not find an alteration in IRS activation. It is therefore possible that HFF diet, administered for only 4 weeks, is able to induce adipose tissue insulin resistance, as well as brain inflammation and oxidative stress [6], but does not trigger impaired response to insulin in brain. Of note, we recently found a decrease in the protein content of insulin degrading enzyme in hippocampus and cortex of middle-aged rats fed an HFF diet (data not shown). Thus, the impairment in the pathway of degradation of insulin in the brain could act as a potentiating factor on the action of this hormone.

The increased activation of the Erk pathway in the hippocampus could be envisaged as marker of damage of this brain area. In fact, Erk activation seems to play an active role in several models of neuronal death, such as hyperglycaemia-mediated neuronal damage [41] or β-amyloid-induced neuronal death [42]. On the other hand, frontal cortex is differently affected, and the decrease in Akt and Erk pathways is likely due to the impairment in other mechanisms, including neurotrophin signaling, since both Akt and Erk are at the crossroad of several intracellular pathways.

The analysis of changes in adiponectin shows a downregulation in e-WAT. This result is in good agreement with lower protein levels of UCP2 and PPAR-α, since it has been shown that adiponectin expression is regulated via adipose PPAR-α [43], and UCP2 controls adiponectin gene expression in adipose tissue [40].

A diet-associated reduction of adiponectin was also observed in both hippocampus and frontal cortex. The origin of adiponectin found in the brain has been debated. Adiponectin can enter the brain by passing through the blood–brain barrier [44]. Hence, the peripheral adipose tissue could be the source of brain adiponectin, even though it has been reported that adiponectin is also produced inside the brain [45]. It is worth mentioning that, beside its role in improving insulin sensitivity and modulating lipid and carbohydrate metabolism, adiponectin was reported to influence neurogenesis, hippocampal synapses and synaptic plasticity [46,47,48]. Furthermore, there is strong evidence supporting the neuroprotective effects of adiponectin in cell culture and animal models [49,50]. Therefore, adiponectin decrease might underlie alteration in brain functioning, as suggested by the reduction in BDNF, a further critical marker of brain functioning, playing a key role in modulation of adult neurogenesis [51] and synaptic function [52]. Our result is in agreement with previous studies showing a downregulation of BDNF signaling both in middle-aged [53] and adolescent [54] rats exposed to a Western diet. Accordingly, we found a significant decrease in the amount of synaptotagmin I in both hippocampus and frontal cortex of middle aged HFF rats. As known, synaptotagmin I participates in the regulation of synaptic vesicle exocytosis, acting for clamping synaptic vesicle fusion in mammalian neurons [22]. Despite a lower level of BDNF in both hippocampus and frontal cortex of the HFF group, no differences in the other markers, synaptophysin and synapsin I, were detected. Whether this is due to the fact that BDNF levels are still enough to maintain downstream targets or to the activation of other signaling pathways remains to be elucidated. Intriguingly, a significant increase in the post-synaptic critical protein PSD-95 was found in both hippocampus and frontal cortex, that could represent a compensatory mechanism against HFF diet-induced presynaptic alterations.

## 4. Materials and Methods

### 4.1. Materials

Bovine serum albumin fraction V (BSA), rabbit anti-human haptoglobin, salts, and buffers were purchased from Sigma-Aldrich (St. Louis, MO, USA). The dye reagent for protein titration was from Bio-Rad (Hercules, CA, USA), and the polyvinylidene difluoride (PVDF) membrane was from GE Healthcare (Milan, Italy). Horseradish peroxidase (HRP)-conjugated secondary antibodies were from Immunoreagent, (Raleigh, NC, USA) (goat anti-rabbit or goat anti-mouse) or from Sigma-Aldrich (St. Louis, MO, USA) (rabbit anti-goat). Fuji Super RX 100 films were from Laboratorio Elettronico Di Precisione (Naples, Italy).

### 4.2. Experimental Design

Male Sprague-Dawley rats were purchased from Charles River (Calco, Como, Italy) and used for the experiments. All rats were caged singly in a temperature-controlled room (23 ± 1 °C) with a 12-h light/dark cycle (06.30–18.30). Treatment, housing, and euthanasia of animals met the guidelines set by the Italian Health Ministry. All experimental procedures involving animals were approved by the “Comitato Etico-Scientifico per la Sperimentazione Animale” of the University of Naples Federico II (260/2015-PR).

For the experiments, we used middle-aged rats (11 months old), that were divided in two groups, each composed of eight rats, that were fed an HFF or LF diet for 4 weeks. The composition of the two diets is reported in Table 1. During the dietary treatment, body weight and food and water intake were monitored daily. At the end of the experimental period, the animals were anaesthetized with sodium Tiopental (40 mg/kg body weight) and euthanized by decapitation, and blood, epididymal white adipose tissue (e-WAT), hippocampus, and frontal cortex were harvested. In particular, hippocampus and frontal cortex were dissected as previously published [6,55]. Samples were then snap frozen in liquid nitrogen and stored at −80 °C for subsequent analyses.

### 4.3. Metabolic Analyses

The blood samples were centrifuged at 1400× *g* for 8 min at 4 °C. After centrifugation at 1400× *g* for 8 min at 4 °C, plasma was isolated and stored at −20 °C until used for determination of substrates and hormones. Plasma glucose concentration was measured by a colorimetric enzymatic method (Pokler Italia, Pontecagnano, Italy). Plasma insulin concentration was measured using an enzyme-linked immunosorbent assay (ELISA) kit (Diametra, Segrate, Italy) in a single assay to avoid interassay variations. HOMA index was calculated as follows: (Glucose (mg/dL) × Insulin (mU/L))/405) [56].

### 4.4. Markers of Inflammation in Plasma, e-WAT, Frontal Cortexok, and Hippocampus

TNF-α concentrations were determined using a rat-specific enzyme linked immunosorbent assay (R&D Systems, Minneapolisok, MN, USA) according to the manufacturer’s instruction.

Haptoglobin concentration in plasma, adipose tissue, frontal cortex, and hippocampus samples was measured by ELISA. Samples were diluted (plasma = 1:9000–1:70,000; adipose tissue, 1:1000–1:30,000; frontal cortex and hippocampus, 1:1000–1:30,000) with coating buffer (7 mM Na_2_CO_3_, 17 mM NaHCO_3_, 1.5 mM NaN_3_, pH 9.6), and aliquots (50 µL) were then incubated in the wells of a microtitre plate (Immuno MaxiSorp; overnight, 4 °C). Washing and blocking were carried out as previously reported [57], then, the wells were incubated (1 h, 37 °C) with 50 µL of rabbit anti-human haptoglobin (1:500 in 130 mM NaCl, 20 mM Tris-HCl, 0.05% Tween, pH 7.4, containing 0.25% BSA), followed by 60 µl of HRP-coniugated secondary antibody (1:5000 dilution). Peroxidase-catalyzed color development from o-phenylenediamine was measured at 492 nm.

### 4.5. Western Blotting

Proteins were extracted from e-WAT by diluting tissue samples 1:1 with lysis buffer (20.0 mmol/L Tris, pH 8, 5% glycerol, 138 mM NaCl, 2.7 mM KCl, 1% NP-40, 5 mM ethylenediaminetetraacetic acid, 5% protease inhibitor cocktail, 1% phosphatase inhibitor cocktail). Homogenates were centrifuged (15,000× *g*, 15 min at 4 °C) and the supernatants were then collected. Aliquots of 20 µg were used for electrophoresis.

Proteins were extracted from hippocampus and frontal cortex by homogenizing aliquots (about 40 mg) of frozen tissues in six volumes (w/v) of cold buffer, as previously published [58]. Homogenates were centrifuged (14,000× *g*, 45 min, 4 °C), and supernatants were then collected. Aliquots of 40 µg were used for electrophoresis.

All the plasma samples were adjusted to protein concentration of 10 µg/µL and 5 µL were used for electrophoresis.

Samples were fractionated by electrophoresis on 12.5% (to quantify BDNF, synaptophysin, synaptotagmin I, adiponectin, lipocalin), or 10% (to quantify synapsin I, PSD-95, TrkB, p-Akt, p-Erk, p-IRS, PGC-1α, PPAR-α, UCP2) polyacrylamide gel, under denaturing and reducing conditions. After electrophoresis, proteins were blotted onto PVDF membrane, essentially as previously reported [6].

The membranes were pre-blocked (1 h, 37 °C) in PBS, 3% bovine albumin serum, 0.3% Tween 20 (blocking PBS), for p-Akt, p-Erk, p-IRS, lipocalin, PGC-1α, PPAR-α, and UCP2 detection, or in 130 mM NaCl, 20 mM Tris-HCl, pH 7.4, 0.05% Tween 20 (T-TBS) containing 5% non-fat milk (blocking TBS) for all other markers. Membranes were then incubated overnight at 4 °C with antibodies at the appropriate dilutions (see Table S1)

Membranes were washed and then incubated for 1 h with the appropriate HRP-conjugated secondary antibodies. For p-Akt, p-Erk, lipocalin, and PPAR-α, the membranes were washed and incubated at room temperature with a chemiluminescent substrate, Immobilon (Millipore Corporation, Billerica, MA 01821, USA). For all the other markers, detection was carried out using the Excellent Chemiluminescent detection Kit (ElabScience, distributed by Microtech, Naples, Italy).

Quantitative densitometry of the bands was carried out by analyzing chemidoc images or digital images of X-ray films exposed to immunostained membranes using Image Lab Software (Biorad Laboratories S.r.l., Segrate (MI)—Italy).

### 4.6. Statistical Analysis

Data were expressed as mean values ± SEM. The program GraphPad Prism 8 (GraphPad Software, San Diego, CA, USA) was used to perform statistical analysis by applying two-tailed, unpaired, Student’s t-test or two-way ANOVA followed by Tukey post-test. *p* < 0.05 was considered significant.

## 5. Conclusions

**Figure 12:**
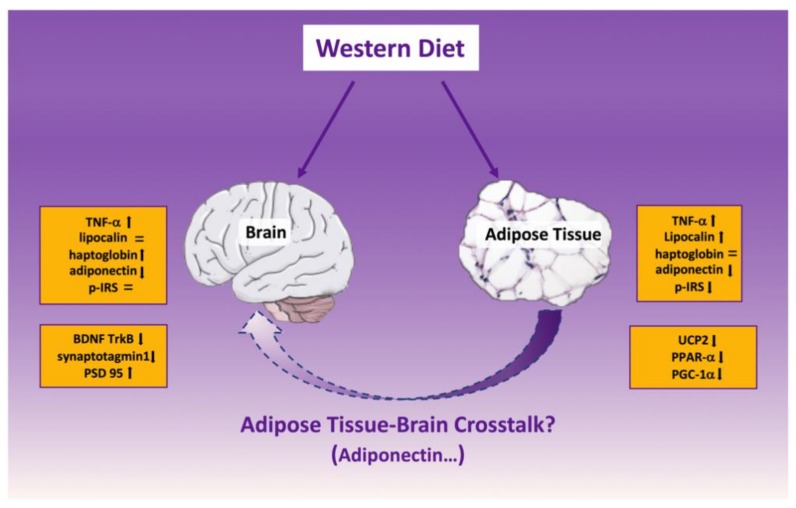
Summary of the modifications induced by high fat-high fructose (western) diet in brain (hippocampus and cortex) and white adipose tissue from middle-aged rats. UCP2=uncoupling protein 2 PPAR-α=peroxisome proliferator activated receptor alpha, PGC-1α=peroxisome proliferator-activated receptor gamma coactivator 1-alpha, TNF-α= tumor necrosis factor alpha, p-IRS=phosphorylated insulin receptor substrate, BDNF=brain derived neurotrophic factor, TrkB=tropomyosin receptor kinase b.

Overall, the general picture of this study (summarized in Figure 12), in middle aged rodent model, shows that the responses to the HFF diet are similar in adipose tissue and brain tissue for several (UCP2, PPAR-α, PGC-1α, TNF-α, adiponectin), but not all the measured parameters (i.e., insulin resistance onset in e-WAT but not brain, or lipocalin and haptoglobin, whose level is not modified in brain or e-WAT, respectively). Whether this similarity is due to crosstalk between the two areas or they are interconnected by a third player is currently unknown and represents an important issue for further studies.

## Figures and Tables

**Table 1:** Diet composition.

	Low Fat	High Fat-High Fructose
**Component, g/1000 g**		
Standard chow ^a^	395.3	231.5
Sunflower oil	19.3	19.3
Casein	59.7	133.3
Water	175.7	175.4
AIN-93 Mineral mix	11.4	11.4
AIN-93 Vitamin mix	3.2	3.2
Choline	0.7	0.7
Methionine	0.9	0.9
Cornstarch	333.8	-------
Butter	-------	129.8
Fructose	-------	294.6
**Energy content and composition**		
ME content, kJ/g ^b^	11.2	14.9
Lipids, J/100 J	10.5	39.3
Proteins, J/100 J	19.9	19.8
Complex carbohydrates, J/100 J	63.9	7.5
Simple sugars, J/100 J	5.7	33.4

^a^ 4RF21, Mucedola, Italy; ^b^ Estimated by computation using values (kJ/g) for energy content as follows: protein 16.736, lipid 37.656, and carbohydrate 16.736. ME = metabolizable energy.

## Supplementary Materials

Supplementary materials can be found at https://www.mdpi.com/1422-0067/21/3/786/s1. Table S1:List of antibodies used for western blot with provider, ref. no. and appropriate dilution.

## References

[1] Hill J.O., Wyatt H.R., Peters J.C. (2012). Energy balance and obesity. Circulation.

[2] Luppino F., de Wit L., Bouvy P.F., Et A. (2010). Overweight, obesity, and depression: A systematic review and meta-analysis of longitudinal studies. Arch. Gen. Psychiatry.

[3] Gustafson D.R. (2012). Adiposity and cognitive decline: Underlying mechanisms. J. Alzheimers Dis..

[4] Arshad N., Lin T.S., Yahaya M.F. (2018). Metabolic syndrome and its effect on the brain: Possible mechanism. CNS Neurol. Disord. Drug Targets.

[5] Cigliano L., Spagnuolo M.S., Crescenzo R., Cancelliere R., Iannotta L., Mazzoli A., Liverini G., Iossa S. (2018). Short-term fructose feeding induces inflammation and oxidative stress in the hippocampus of young and adult rats. Mol. Neurobiol..

[6] Crescenzo R., Spagnuolo M.S., Cancelliere R., Iannotta L., Mazzoli A., Gatto C., Iossa S., Cigliano L. (2019). Effect of Initial Aging and High-Fat/High-Fructose Diet on Mitochondrial Bioenergetics and Oxidative Status in Rat Brain. Mol. Neurobiol..

[7] Bomfim T.R., Forny-Germano L., Sathler L.B., Brito-Moreira J., Houzel J.C., Decker H., Silverman M.A., Kazi H., Melo H.M., McClean P.L. (2012). An anti-diabetes agent protects the mouse brain from defective insulin signaling caused by Alzheimer’s disease–associated Ab oligomers. J. Clin. Invest..

[8] Lourenco M.V., Clarke J.R., Frozza R.L., Bomfim T.R., Forny-Germano L., Batistam A.F., Sathler L.B., Brito-Moreira J., Amaral O.B., Silva C.A. (2013). TNF-α mediates PKR-dependent memory impairment and brain IRS-1 inhibition induced by Alzheimer’s b-amyloid oligomers in mice and monkeys. Cell Metab..

[9] De Felice F.G., Ferreira S.T. (2014). Inflammation, defective insulin signaling, and mitochondrial dysfunction as common molecular denominators connecting type 2 diabetes to Alzheimer disease. Diabetes.

[10] Ogden C.L., Carroll M.D., Fryar C.D., Flegal K.M. (2015). Prevalence of obesity among adults and youth: United States, 2011–2014. NCHS Data Briefs.

[11] Kivipelto M., Ngandu T., Fratiglioni L., Viitanen M., Kåreholt I., Winblad B., Helkala E.L., Tuomilehto J., Soininen H., Nissinen A. (2005). Obesity and vascular risk factors at midlife and the risk of dementia and Alzheimer disease. Arch. Neurol..

[12] Whitmer R.A., Gunderson E.P., Barrett-Connor E., Quesenberry C.P., Yae K. (2005). Obesity in middle age and future risk of dementia: A 27-year longitudinal population-based study. BMJ.

[13] Mazzoli A., Crescenzo R., Cigliano L., Spagnuolo M.S., Cancelliere R., Gatto C., Iossa S. (2019). Early hepatic oxidative stress and mitochondrial changes following western diet in middle aged rats. Nutrients.

[14] Della Corte K.W., Perrar I., Penczynski K.J., Schwingshackl L., Herder C., Buyken A.E. (2018). Effect of dietary sugar intake on biomarkers of subclinical inflammation: A systematic review and meta-analysis of intervention studies. Nutrients.

[15] Duan Y., Zeng L., Zheng C., Song B., Li F., Kong X., Xu K. (2018). Inflammatory links between high fat diets and diseases. Front Immunol..

[16] Esmaili S., Xu A., George J. (2014). The multifaceted and controversial immunometabolic actions of adiponectin. Trends Endocrinol. Metab..

[17] Bloemer J., Pinky P.D., Govindarajulu M., Hong H., Judd R., Amin R.H., Moore T., Dhanasekaran M., Reed M.N., Suppiramaniam V. (2018). Role of adiponectin in central nervous system disorders. Neural. Plast..

[18] Maffei M., Barone I., Scabia G., Santini F. (2016). The multifaceted haptoglobin in the context of adipose tissue and metabolism. Endocr. Rev..

[19] Zhang J., Wu Y., Zhang Y., LeRoith D., Bernlohr D.A., Chen X. (2008). The role of lipocalin 2 in the regulation of inflammation in adipocytes and macrophages. Mol. Endocrinol..

[20] Kowiański P., Lietzau G., Czuba E., Waśkow M., Steliga A., Moryś J. (2018). BDNF: A key factor with multipotent impact on brain signaling and synaptic plasticity. Cell Mol. Neurobiol..

[21] Cesca F., Baldelli P., Valtorta F., Benfenati F. (2010). The synapsins: Key actors of synapse function and plasticity. Prog. Neurobiol..

[22] Courtney N.A., Bao H., Briguglio J.S., Chapman E.R. (2019). Synaptotagmin 1 clamps synaptic vesicle fusion in mammalian neurons independent of complexin. Nat. Commun..

[23] Chen X., Nelson C.D., Li X., Winters C.A., Azzam R., Sousa A.A., Leapman R.D., Gainer H., Sheng M., Reese T.S. (2011). PSD-95 is required to sustain the molecular organization of the postsynaptic density. J. Neurosci..

[24] Anstey K.J., Cherbuin N., Budge M., Young J. (2011). Body mass index in midlife and late life as a risk factor for dementia: A meta-analysis of prospective studies. Obes. Rev..

[25] Jauch-Chara K., Oltmanns K.M. (2014). Obesity a neuropsychological disease? Systematic review and neuropsychological model. Prog. Neurobiol..

[26] Xu W.L., Atti A.R., Gatz M., Pedersen N.L., Johansson B., Fratiglioni L. (2011). Midlife overweight and obesity increase late-life dementia risk: A population-based twin study. Neurology.

[27] Dandona P., Aljada A., Chaudhuri A., Mohanty P., Garg R. (2005). Metabolic syndrome: A comprehensive perspective based on interactions between obesity, diabetes, and inflammation. Circulation.

[28] Bhat N.R. (2010). Linking cardiometabolic disorders to sporadic Alzheimer’s disease: A perspective on potential mechanisms and mediators. J. Neurochem..

[29] Parimisetty A., Dorsemans A.C., Awada R., Ravanan P., Diotel N., Lefebvre d’Hellencourt C. (2016). Secret talk between adipose tissue and central nervous system via secreted factors—an emerging frontier in the neurodegenerative research. J. Neuroinflamm..

[30] Huffman D.M., Barzilai N. (2009). Role of visceral adipose tissue in aging. Biochim. Biophys. Acta Gen. Subj..

[31] Sutinen E.M., Pirttilä T., Anderson G., Salminen A., Ojala J.O. (2012). Pro-inflammatory interleukin-18 increases Alzheimer’s disease associated amyloid-β production in human neuron-like cells. J. Neuroinflamm..

[32] Chusyd D.E., Wang D., Huffman D.M., Nagy T.R. (2016). Relationships between rodent white adipose fat pads and human white adipose fat depots. Front. Nutr..

[33] Guo H., Bazuine M., Jin D., Huang M.M., Cushman S.W., Chen X. (2013). Evidence for the regulatory role of lipocalin 2 in high-fat diet-induced adipose tissue remodeling in male mice. Endocrinology.

[34] Takahashi H., Sanada K., Nagai H., Li Y., Aoki Y., Ara T., Seno S., Matsuda H., Yu R., Kawada T. (2017). Over-expression of PPARα in obese mice adipose tissue improves insulin sensitivity. Biochem. Biophys. Res. Commun..

[35] Cheng C.F., Ku H.C., Lin H. (2018). PGC-1α as a pivotal factor in lipid and metabolic regulation. Int. J. Mol. Sci..

[36] Chevillotte E., Giralt M., Miroux B., Ricquier D., Villarroya F. (2007). Uncoupling protein-2 controls adiponectin gene expression in adipose tissue through the modulation of reactive oxygen species production. Diabetes.

[37] Shoelson S.E., Herrero L., Naaz A. (2007). Obesity, Inflammation, and Insulin Resistance. Gastroenterol.

[38] Yan Q.W., Yang Q., Mody N., Graham T.E., Hsu C.H., Xu Z., Houstis N.E., Kahn B.B., Rosen E.D. (2007). The Adipokine Lipocalin 2 Is Regulated by Obesity and Promotes Insulin Resistance. Diabetes.

[39] Salvatore A., Cigliano L., Bucci E.M., Corpillo D., Velasco S., Carlucci A., Pedone C., Abrescia P. (2007). Haptoglobin binding to apolipoprotein A-I prevents damage from hydroxyl radicals on its stimulatory activity of the enzyme lecithin-cholesterol acyl-transferase. Biochemistry.

[40] Maresca B., Spagnuolo M.S., Cigliano L. (2015). Haptoglobin modulates beta-amyloid uptake by U-87MG astrocytes cell line. J. Mol. Neurosci..

[41] Zhang J.Z., Jing L., Guo F.Y., Ma Y., Wang Y.L. (2007). Inhibitory effect of ketamine on phosphorylation of the extracellular signal-regulated kinase 1/2 following brain ischemia and reperfusion in rats with hyperglycemia. Exp. Toxicol. Pathol..

[42] Frasca G., Carbonaro V., Merlo S., Copani A., Sortino M.A. (2008). Integrins mediate beta-amyloid-induced cell-cycle activation and neuronal death. J. Neurosci. Res..

[43] Goto T., Lee J.Y., Teraminami A., Kim Y.I., Hirai S., Uemura T., Inoue H., Takahashi N., Kawada T.J. (2011). Activation of peroxisome proliferator-activated receptor-alpha stimulates both differentiation and fatty acid oxidation in adipocytes. Lipid Res..

[44] Thundyil J., Pavlovski D., Sobey C.G., Arumugam T.V. (2012). Adiponectin receptor signalling in the brain. Br. J. Pharm..

[45] Wilkinson M., Brown R., Imran S.A., Ur E. (2007). Adipokine gene expression in brain and pituitary gland. Neuroendocrinology.

[46] Zhang D., Wang X., Lu X.Y. (2016). Adiponectin exerts neurotrophic effects on dendritic arborization, spinogenesis, and neurogenesis of the dentate gyrus of male mice. Endocrinology.

[47] Zhang D., Guo M., Zhang W., Lu X.Y. (2011). Adiponectin stimulates proliferation of adult hippocampal neural stem/progenitor cells through activation of p38 mitogen-activated protein kinase (p38MAPK)/glycogen synthase kinase 3β (GSK-3β)/β-catenin signaling cascade. J. Biol. Chem..

[48] Yau S.Y., Li A., Hoo R.L., Ching Y.P., Christie B.R., Lee T.M., Xu A., So K.F. (2014). Physical exercise-induced hippocampal neurogenesis and antidepressant effects are mediated by the adipocyte hormone adiponectin. Proc. Natl. Acad Sci. USA.

[49] Letra L., Rodrigues T., Matafome P., Santana I., Seiça R. (2017). Adiponectin and sporadic Alzheimer’s disease: Clinical and molecular links. Front Neuroendocr..

[50] Chan K.H., Lam K.S., Cheng O.Y., Kwan J.S., Ho P.W., Cheng K.K., Chung S.K., Ho J.W., Guo V.Y., Xu A. (2012). Adiponectin is protective against oxidative stress induced cytotoxicity in amyloid-beta neurotoxicity. PLoS ONE.

[51] Scharfman H., Goodman J., Macleod A., Phani S., Antonelli C., Croll S. (2005). Increased neurogenesis and the ectopic granule cells after intrahippocampal BDNF infusion in adult rats. Exp. Neurol..

[52] Leal G., Afonso P.M., Salazara I.L., Duarte C.B. (2015). Regulation of hippocampal synaptic plasticity by BDNF. Brain Res..

[53] Stranahan A.M., Norman E.D., Lee K., Cutler R.G., Telljohann R.S., Egan J.M., Mattson M.P. (2008). Diet-induced insulin resistance impairs hippocampal synaptic plasticity and cognition in middle-aged rats. Hippocampus.

[54] Hussain Y., Jain S.K., Samaiya P.K. (2019). Short-term westernized (HFFD) diet fed in adolescent rats: Effect on glucose homeostasis, hippocampal insulin signaling, apoptosis and related cognitive and recognition memory function. Behav. Brain Res..

[55] Spagnuolo M.S., Bergamo P., Crescenzo R., Iannotta L., Treppiccione L., Iossa S., Cigliano L. (2018). Brain Nrf2 pathway, autophagy, and synaptic function proteins are modulated by a short-term fructose feeding in young and adult rats. Nutr. Neurosci..

[56] Cacho J., Sevillano J., de Castro J., Herrera E., Ramos M.P. (2008). Validation of simple indexes to assess insulin sensitivity during pregnancy in Wistar and Sprague-Dawley rats. Am. J. Physiol. Endocrinol. Metab..

[57] Spagnuolo M.S., Mollica M.P., Maresca B., Cavaliere G., Cefaliello C., Trinchese G., Scudiero R., Crispino M., Cigliano L. (2015). High Fat Diet and Inflammation—Modulation of Haptoglobin Level in Rat Brain. Front Cell. Neurosci..

[58] Spagnuolo M.S., Maresca B., Mollica M.P., Cavaliere G., Cefaliello C., Trinchese G., Esposito M.G., Scudiero R., Crispino M., Abrescia P. (2014). Haptoglobin increases with age in rat hippocampus and modulates Apolipoprotein E mediated cholesterol trafficking in neuroblastoma cell lines. Front. Cell. Neurosci..

