# Supplementary material for: Adipose Tissue and Brain Metabolic Responses to Western Diet—Is There a Similarity between the Two?
Source: Int J Mol Sci. 2020 Jan 25;21(3):786. doi: 10.3390/ijms21030786 (PMC7036881; doi:10.3390/ijms21030786)
Supplement: Supplementary file 1 [file ijms-21-00786-s001.pdf]

Table S1: List of antibodies used for western blot with provider, ref. no. and appropriate dilution.

Provider	Antigen	Ref. no.	Dilution
Calbiochem Merck			
	**UCP2**	144-157	1: 1000
Cell Signaling Technology			
	**pIRS**	2388	1: 1000
	**IRS**	3407	1: 1000
	**pAkt**	9271	1: 1000
	**Akt**	9272	1: 1000
	**pErk**	4370	1: 1000
	**Erk**	4695	1: 1000
	**synaptotagmin I**	14558	1: 1000
	**PSD-95**	2507	1: 1000
Genetex			
	**synapsin I**	GTX131233	1: 500
Immunological Sciences			
	**Adiponectin**	AB-84172	1: 500
Merk Millipore			
	**PGC-1α**	AB-3242	1: 500
	**Synaptophysin**	AB9272	1: 200000
Santa Cruz Biotechnology			
	**BDNF**	SC546	1: 500
	**TrkB**	sc-377218	1: 2000
Sigma Aldrich			
	**Actin**	A2228	1: 1000
Thermo Fisher Scientific			
	**PPAR-α**	PA1-32484	1: 1000
	**Lipocalin**	PA5-46938	1: 200
